# Assessment of Treatment Response Following Yttrium-90 Transarterial Radioembolization of Liver Malignancies

**DOI:** 10.7759/cureus.2895

**Published:** 2018-06-29

**Authors:** Charles S Adcock, Edward Florez, Kevin A Zand, Akash Patel, Candace M Howard, Ali Fatemi

**Affiliations:** 1 Radiology, University of Mississippi Medical Center, Jackson, USA; 2 Interventional Radiology, University of Mississippi Medical Center, Jackson, USA; 3 Radiation Oncology, University of Mississippi Medical Center, Jackson, USA

**Keywords:** y90 transarterial radioembolization, brachytherapy, liver cancer, radiation therapy, mri, fdg-pet, post-treatment evaluation, tumour necrosis, tumour vascularity, malignant tumour

## Abstract

Transarterial radioembolization using yttrium-90 microspheres is an established and effective treatment for liver malignancies. Determining response to this treatment is difficult due to the radical changes that occur in tissue as a response to radiation. Though accurate assessment of treatment response is paramount for proper patient disposition, there is currently no standardized assessment protocol. Current methods of assessment often consider changes in size, necrosis, vascularity, fluorodeoxyglucose-positron emission tomography FDG-PET metabolic activity, and diffusion using diffusion-weighted magnetic resonance imaging (DWI). Current methods of assessment require a lag time of one to two months post-treatment to determine treatment effectiveness. This delay is a hindrance to obtaining better patient outcomes, giving rise to a need to identify markers for faster determination of treatment efficacy.

## Introduction and background

Primary malignancies of the liver and intrahepatic bile duct, the most common type being hepatocellular carcinomas (HCC), make up an estimated 2.3% of all new cancer cases and are estimated to be the fifth leading cause of cancer death in men in the United States in2016 [1]. Patients diagnosed with liver cancer have a one-year relative survival rate of 44% and a five-year relative survival rate of 17%. If diagnosed with a localized stage of cancer—a group that includes only 43% of liver cancer patients—the five-year relative survival rate increases to 31% [1]. Worldwide, the median age at diagnosis is 64 years of age, with an average of 16.4 years of life lost per person dying from primary liver malignancies. The most common risk factors for primary liver cancer in the United States are hepatitis B and/or hepatitis C infection. Alcohol abuse, diabetes, obesity, smoking, and genetic disorders such as hemochromatosis are also known risk factors for primary liver cancer [2]. 

Secondary malignancies, or metastases, of the liver are much more common than primary malignancies; the liver is one of the most common sites for metastatic lesions [2]. The most common sources of metastasis are cancers of the breast (an estimated 29% of all cancer cases in women), lung and bronchus (an estimated 13.3% of all new cancer cases), and colorectal tract (an estimated 8% of all new cancer cases) [1]. It is thought that the dual blood supply from both the portal vein and hepatic artery in combination with the easily penetrated fenestrated capillaries and sinusoids of the liver contribute to this increased likelihood of metastasis [2].

Patients who present with malignancies of the liver have a variety of treatment options. Depending on medical comorbidities, the number and size of liver lesions, and stage, various treatment strategies are available, such as systemic chemotherapy, resection, transplantation, ablation, radioembolization, immunoembolization, and/or chemoembolization. Surgical resection and transplantation are the most effective therapies for liver malignancies and produce the best outcomes for those who qualify. Unfortunately, the majority of patients in need of a liver transplant have lesions that are non-resectable, or do not qualify for surgery [3].

For patients not able to undergo resection or transplantation, transarterial radioembolization (TARE) with yttrium-90 (Y-90) microspheres is an increasingly popular treatment option. TARE is performed by image-guided deposition of radiated Y-90 microspheres to the tumor through the hepatic artery, and is the most common source of perfusion for liver metastases and hepatocellular carcinomas [3-4]. TARE aims to deliver a dose of 100–600 Gy local band radiation (brachytherapy) to the tumor while keeping the radiation exposure of normal liver tissue below 40 Gy [5-7].

There are two Y-90 microsphere products commercially available: Therasphere (MDS Nordion, Ottawa, Ontario, Canada) and SIR-Spheres (Sirtex Medical, Lane Cove, Australia). Therasphere microspheres are made of a non-biodegradable glass with a diameter of 20 to 30 µm. SIR-Spheres microspheres are made of a biodegradable resin with a diameter of 35 µm [8].

The assessment of tumor response to TARE treatment is challenging. Measuring only size, as in the Response Evaluation Criteria in Solid Tumors (RECIST) framework, has proven to not accurately measure tumor response to TARE [9]. Current pre- and post-treatment imaging techniques performed for treatment assessment utilize both anatomic cross-sectional imaging such as computed tomography (CT) and magnetic resonance imaging (MRI) combined with functional imaging, such as single-photon emission CT (SPECT), positron emission tomography (PET), dynamic contrast-enhanced (DCE) MRI and/or CT, and diffusion-weighted MRI (DWI). Utilizing both anatomic and functional imaging allows for a more accurate assessment of tumor response [9]. 

## Review

Patient presentation

Secondary malignancies are often asymptomatic and can be detected with anatomic cross-sectional imaging. Hormonally active metastases can present with symptoms related to the hormone they secrete [10]. Primary malignancies are subtle and more likely to present with signs of liver disease like nausea, abdominal pain, weight loss, and rarely, jaundice. Liver function may be abnormal, but often indistinguishable from that of a cirrhotic liver. Signs of portal hypertension such as ascites, varicocele, splenomegaly, and hepatomegaly may also be present and related to the underlying etiology of the primary malignancy. Hematemesis can be present if esophageal varices rupture [11]. 

Patient evaluation

A pretreatment evaluation is required to determine whether a patient qualifies for TARE treatment. The evaluation includes the patient's medical and surgical history, physical exam, an Eastern Cooperative Oncology Group (ECOG) performance status score, and laboratory tests. The medical histories and surgical histories of patients with liver malignancies are often extensive. Laboratory findings should show a granulocyte count of greater than 1.5 x 109/L, platelet count greater than or equal to 50 x 109/L, creatinine of less than or equal to 2.0 mg/dL, and bilirubin of less than 2.0 mg/dL. Biologic tumor markers (e.g., a-fetoprotein, carcinoembryonic antigen, CA19-9) should also be measured [12]. 

Pretreatment imaging

Anatomic imaging studies required for pretreatment evaluation include chest CT and either CT or MRI of the abdomen. MRI is the standard of care, but those who cannot undergo MRI (claustrophobic patients, or patients with implants) should undergo CT [11]. Contrast-enhanced multiphasic CT is an established method of detecting and characterizing liver lesions [10]. HCC is often hypervascular and can be identified by observing arterial phase enhancement with venous phase washout on multiphase imaging. This makes assessment of HCC vascularity useful in post-treatment assessment. Cholangiocarcinoma, on the other hand, is often hypovascular, and so enhancement is not generally observed in post-TARE imaging [11]. Metastases vary depending on their origin. These variations are another factor that complicate post-TARE imaging [13]. 

**Figure 1:**
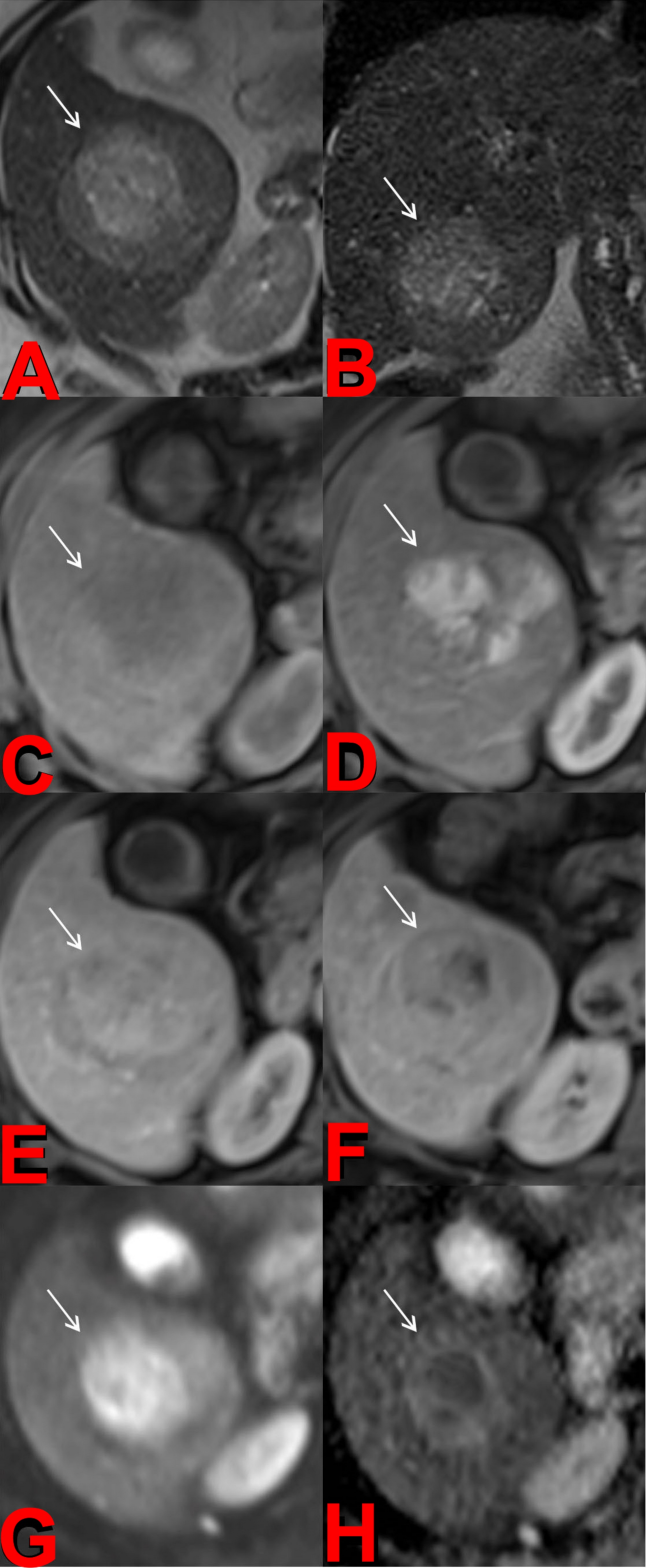
Pretreatment Magnetic Resonance Imaging (MRI) Axial (a) and coronal (b) T2-weighted images of the liver demonstrate a mildly hyperintense mass in the right hepatic lobe. Precontrast (c), arterial (d), portalvenous (e), and delayed (f) postcontrast T1-weighted images demonstrate heterogeneous early arterial hyperenhancement and subsequent portalvenous / delayed washout of the mass compatible with hepatocellular carcinoma. The diffusion weighted imaging (DWI) (g) and its corresponding apparent diffusion coefficient (ADC) map (h) demonstrate foci of restricted diffusion within the tumor.

DCE-MRI is proven to be only a slightly better diagnostic tool than contrast-enhanced CT [9]. One advantage of MRI is that it shows a superior ability to detect smaller lesions. The biggest advantage of MRI, though, is the additional functional imaging technique of DWI, which can be used to determine the cellularity and apparent diffusion coefficient (ADC) of tissue. Cellularity and ADC can help differentiate many solid malignant lesions from benign cystic lesions (Figure 1) [10].

**Figure 2:**
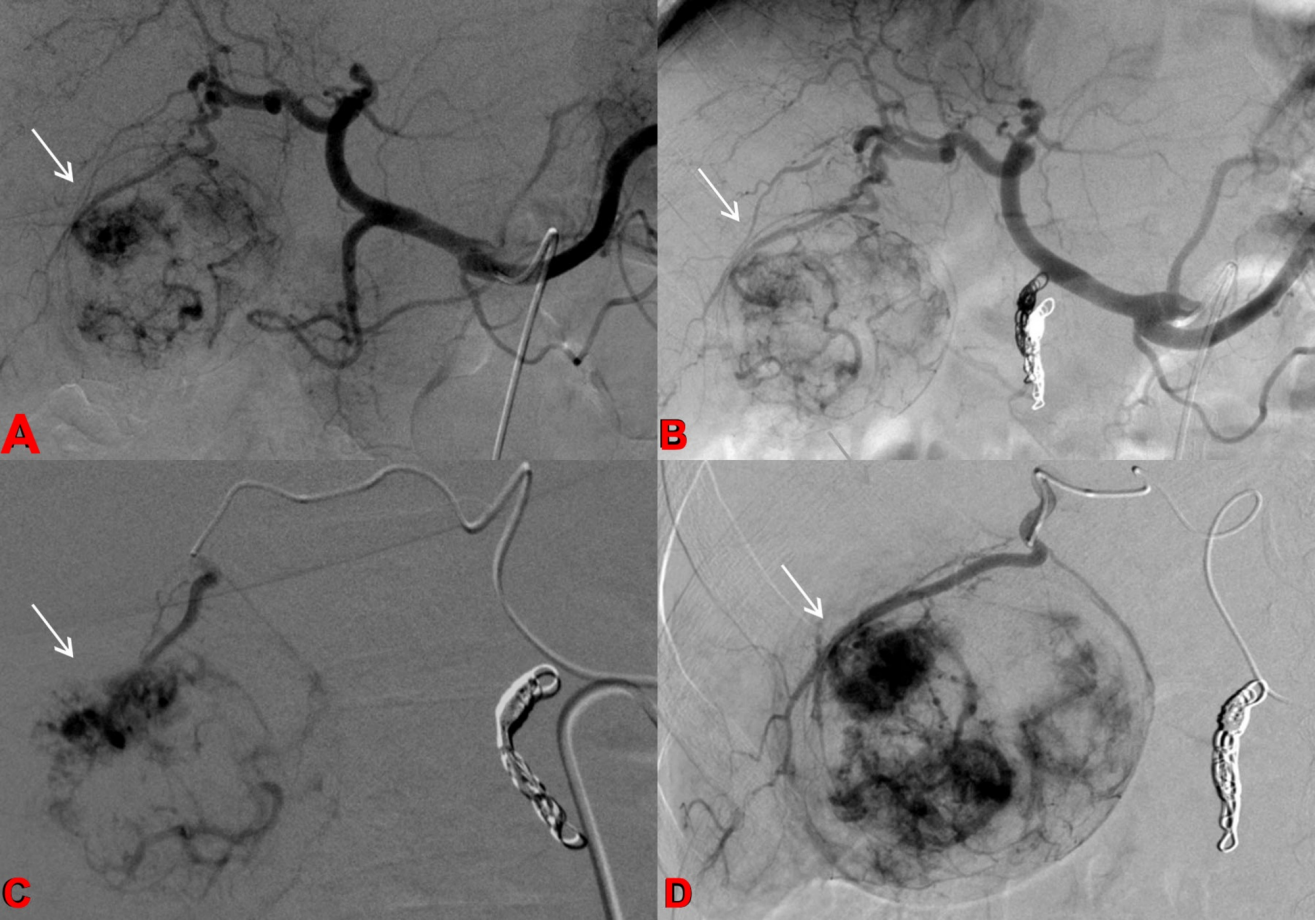
Pretreatment Angiogram Celiac arteriograms before and after embolization of the gastroduodenal artery (a-b) demonstrate conventional celiac vascular anatomy with tumor blush in the right hepatic lobe corresponding to the known hepatocellular carcinoma (HCC). C-D: Selective arteriograms of the hepatic arterial branches supplying the hypervascular HCC.

Angiography of the abdominal aorta, superior mesenteric artery, and celiac artery is performed to assess for anatomic variants, map the visceral anatomy, and evaluate the tumor blood supply. Before treatment can be performed, embolization of the extrahepatic arteries is necessary to restrict the radiated microspheres from non-targeted dispersion to undesired locations outside the tumor bed (Figure 2) [14].

**Figure 3:**
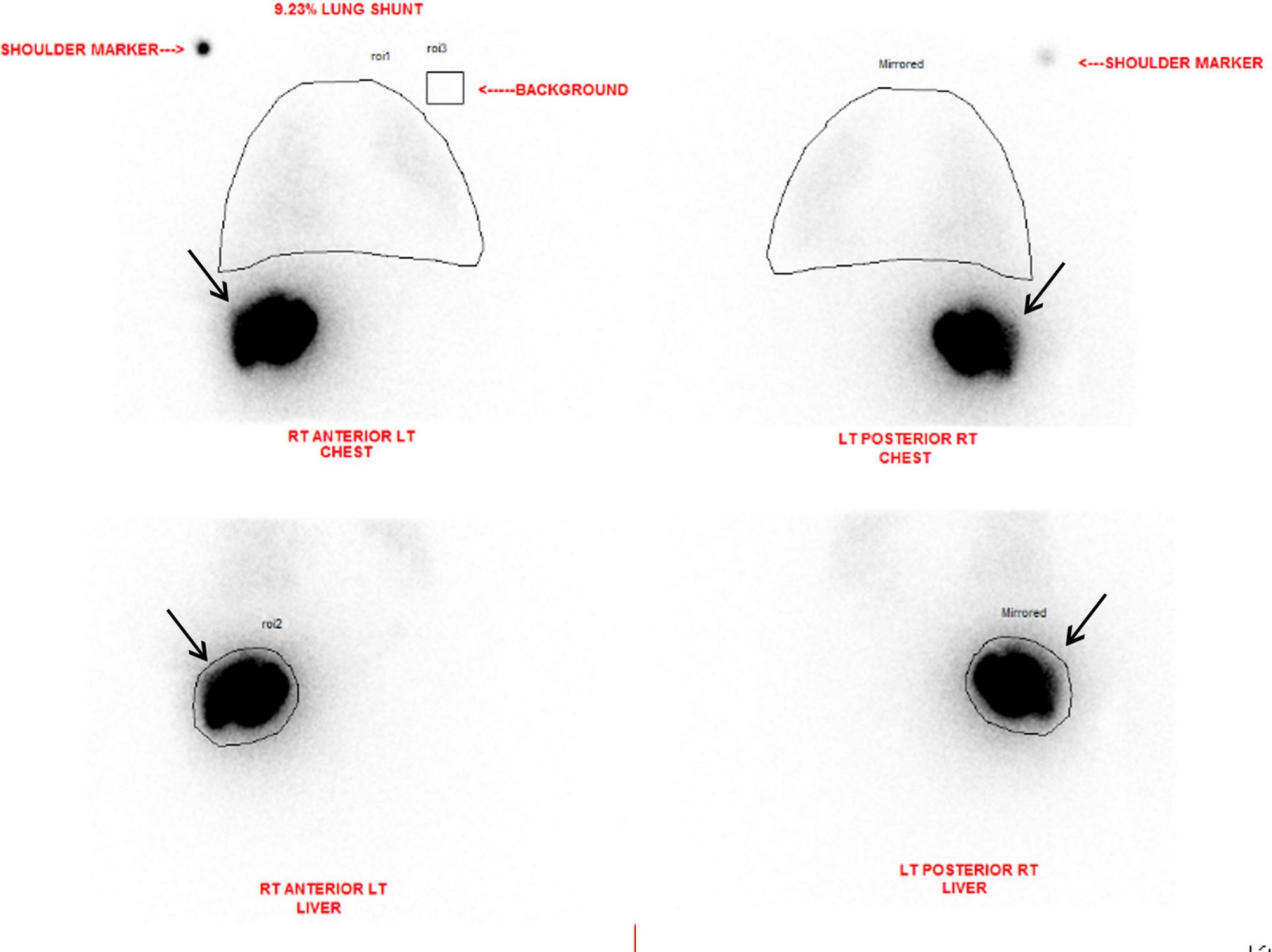
Liver-Lung Perfusion Scan Anterior and posterior planar imaging of both chest and abdomen were performed after selective arterial administration of 5 mCi of Tc-99m MAA by the interventional radiologist. Region of interests (ROIs) were placed over the liver and lungs. The planar images demonstrate activity in the right lobe of the liver and no significant activity in the lungs or bowel. Mean calculated lung shunting is 9.23 %.

Technetium-99m (99mTc) macroaggregated albumin (MAA) SPECT imaging is often performed prior to TARE (Figure 3). The intrahepatic and extrahepatic distributions of the tracer are examined, and the resulting is data used to calculate dosage delivery. Lung shunt fractions are also calculated from the SPECT imaging. Patients with a significant amount of non-correctable shunting to other extrahepatic tissues, such as the gastrointestinal tract, should also be excluded [14].

Dosimetry

The success of TARE depends on the prescribed amount of radiation being delivered to the target lesion. Determining the dosage of radiation that will be delivered to the patient is a vital step in the TARE procedure. TARE dosimetry considers the patient’s body mass index along with the volume of the liver lobe or segment that is to receive treatment. Willowson et al. analyzed dosage delivery with Y-90 PET/CT studies conducted within 24 hours after TARE treatment in 22 patients with 63 colorectal liver metastases; they concluded that lesions receiving > 50 Gy were more likely to produce a significant response to treatment. They also found that dose heterogeneity was a significant prognostic factor for lesions receiving < 50 Gy. When dose heterogeneity was combined with average dosage delivery, it had a positive predictive value of > 80%; if a lesion received < 20 Gy it was unlikely to respond to treatment [15]. If dosage delivery to target lesions correlates with treatment success, it may have potential as an immediate indicator of treatment success. 

Tumor assessment in post-treatment imaging 

A standard protocol for post-TARE imaging does not currently exist. CT, PET/CT, and/or MRI are used at varying times at the discretion of the institution directing treatment. Post-TARE imaging generally begins a month after treatment and is repeated every two to three months thereafter. Boas et al. suggest that the optimal scheduling for post-treatment imaging is at 2, 4, 6, 8, 11, 14, 18, and 24 months. The high frequency of scanning in the first year after treatment is warranted by the 6.5x greater chance of recurrent disease in that time span [16].

Multiple criteria are used to determine tumor response to treatment. Many tumor evaluations utilize changes in size as the primary biomarker for success, but an initial increase in tumor size does not necessarily mean tumor progression following a radioembolization treatment [17]. An apparent increase in tumor size can be caused by the radiated microspheres reshaping the environment in which they are distributed, among other factors. 

This creates a variety of challenges for those assessing tumor response with imaging modalities. Singh and Anil describe many findings common in post-treatment images in their work [9]. Common findings include peritumoral edema, hemorrhage, ring enhancement, biliary complications, abscess, radiation-induced liver disease, non-targeted radioembolization, perihepatic ascites, pleural effusion, capsular retraction, hepatic lobar volumetric changes, fibrosis, and portal hypertension. Changes in tumor size, necrosis, vascularity, metabolic activity, and cellularity as seen in baseline and post-treatment imaging can be used to assess tumor response. Current practice relies most heavily on changes in size and vascularity to track tumor response [9].

Changes in Tumor Size

While it may take longer to occur in TARE, changes in tumor size are the ultimate indicator of tumor response. Anatomic CT and/or MRI are most commonly used to measure tumor size. Measuring the maximum diameter, as in the non-modified Response Evaluation Criteria in Solid Tumors (RECIST) guidelines [3]; measuring the cross product of the maximum diameter and the maximum perpendicular dimension, as in the World Health Organization (WHO) guidelines for tumor assessment; or measuring tumor volume have been the most popular pre- and post-treatment methods for assessing tumor size [4]. Due to high measurement variability between the first two parameters, tumor volume is the most reliable measurement for determining reductions in tumor size [9]. Partial response is defined as a 65% or greater reduction in tumor volume [12,18]. 

Necrosis

Tumor necrosis, hemorrhage, and edema caused by the treatment can contribute to an initial increase in tumor size after treatment. Keppke et al. were the first to suggest considering necrosis in treatment assessments for TARE [4]. An increase in tumor size with no enhancement is often seen around 30 days post-TARE treatment. This increased size may persist for months. In tumors responding to treatment, a change in size is generally seen after about 119 days, while a necrosis response is seen after 29–30 days. A combined approach of measuring tumor size and necrosis yields response around 31–34 days [4,9,19]. 

The European Association for the Study of the Liver (EASL) criteria for HCC includes a recommendation that estimates of a viable tumor be based on contrast-enhanced imaging. The American Association for the Study of Liver Disease (AASLD) endorses the EASL criteria, and the RECIST assessment criteria have also been modified (mRECIST) to follow suit, to account for viable tumor and necrosis [9].

**Figure 4:**
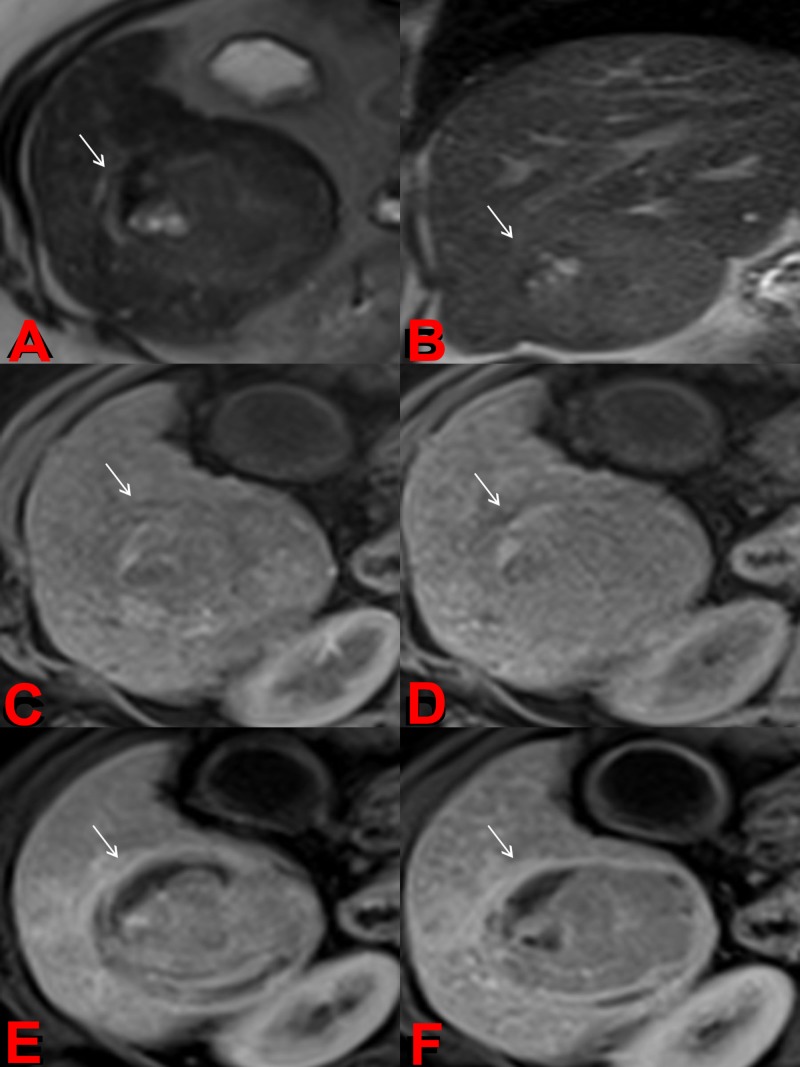
Post-treatment Magnetic Resonance Imaging (MRI) Axial (a) and coronal (b) T2-weighted images demonstrate an overall decrease in signal intensity of treated HCC with interval development of hyperintense foci compatible with cystic necrosis. Precontrast (c), arteral (d), portalvenous (e), and delayed (f) postcontrast T1-weighted images demonstrate lack of tumor enhancement compatible with nonviable tumor and necrosis.

Necrosis is not always complete and may frequently present in patches. These changes are seen between seven and thirty days post-TARE and may persist for months. These findings do not possess any predictive value during the first 90 days after treatment. If, however, these findings persist for more than 90 days, it is likely residual disease, especially if seen with arterial phase enhancement (e.g., Figure 4) [9,20].

Diffusion-Weighted MR Imaging

Detecting tumor size may be difficult due to common post-treatment findings such as edema. These benign findings can be detected using DWI, which is particularly useful for detecting the presence of hypovascular tumors. DWI can detect the diffusion of water molecules, providing information vital for tumor assessment. Areas allowing locally increased diffusion of water molecules may indicate decreased cellularity and compromised cellular membrane integrity in necrotic tissues. DWI presents this information as an apparent diffusion coefficient (ADC). A retrospective study of 150 patients with 153 hepatic lesions performed by Parsai et al. found that the ADC values of edema, necrosis, and cysts are higher than those of HCC and metastases; therefore, DWI can be useful in differentiating the amount of solid tumor remaining from benign findings in tumor assessment [21]. ADC values have been used to determine accurate tumor response within 42 days post-TARE [9,22].

Vascularity

All patients receiving TARE undergo a preliminary angiography to examine the extent of the perfusion of the liver. This is intended to identify variant vasculature that may deliver Y-90 microspheres to non-target tissues [12]. Since Y-90 is delivered through the hepatic artery, the vasculature supplying the tumor can potentially be destroyed; most liver tumors, including HCC and metastases, receive most of their blood supply directly from the hepatic artery. Damage to the hepatic artery does not pose a major threat to normal liver tissue since the normal liver parenchyma receives 75% of its perfusion from the hepatic portal vein [20]. 

On CT, non-enhancing lesions are not the only indication of reduced vascularity. Lesions showing a similar enhancement to normal liver parenchyma may be considered a favorable response for more hypervascular lesions. The complete disappearance of a tumor may indicate a loss of vasculature for smaller lesions [9,20]. Parsai et al. found no difference between the ADC values of hypovascular and hypervascular malignant lesions in their retrospective study [21]. This further suggests that DWI may play a valuable role in the routine monitoring of hepatic lesions.

A retrospective study by Zhu et al. of 14 patients with hypovascular metastatic lesions to the liver treated with TARE showed a significant decrease in arterial and venous enhancement, and a significant increase in volumetric ADC in 21 responding lesions by RECIST criteria when examined with contrast-enhanced MRI one month after treatment. Responding lesions, however, lacked significant changes in size when evaluated with anatomic imaging. They concluded that RECIST, mRECIST, and EASL were inadequate in the assessment of post-TARE imaging of hypovascular liver metastasis. They suggested that a quantitative volumetric functional MRI should be performed in future research and clinical trials, because it may predict outcomes earlier than the currently used criteria [23]. Sobhani et al. reported similar conclusions in a retrospective study of 17 patients who underwent transarterial chemoembolization (TACE), a similar embolizing therapy that uses chemotherapy drugs and microspheres as opposed to irradiated microspheres. They stated that a volumetric contrast-enhanced and diffusion-weighted MRI may prove effective in the early evaluation of treatment response in hypovascular lesions [24].

Changes in FDG-PET Metabolic Activity

PET analysis is standard practice for post-treatment assessment of various cancers. FDG-PET/CT depicts the metabolic activity of malignant tissue. Unfortunately, FDG-PET/CT is not as effective at detecting hepatocellular carcinoma as contrast-enhanced CT, since well-differentiated HCC does not accumulate FDG [7]. FDG-PET/CT improves tumor response data when compared to just anatomic cross-sectional imaging for metastases [9, 19]. FDG-PET/CT has a higher capability to differentiate benign post-TARE findings from residual liver metastases in radiofrequency ablation [9,25]. However, FDG-PET/CT is not often routinely used in post-TARE follow-up due to it being an expensive and resource-intense modality [9].

Discussion

Determining the most accurate assessment modality for tumor response to TARE treatment is vital for patient care, because the assessment is the foundation for future treatment decisions. An inaccurate assessment can lead to unnecessary or inadequate treatment leading to increased patient morbidity and poor patient outcomes. Current assessment is best performed utilizing both anatomic (CT and MRI) and functional (DCE, DWI, SPECT, and PET) imaging data due to the many challenging findings that arise from TARE.

While multimodal (functional and anatomic) imaging techniques are useful for planning and assessment in radiation therapy, assessing these separate modalities together can be challenging. There are changes and uncertainties inherent in their technical and clinical implementation, such as validating different techniques of registration, fusion, delineation of target and possible critical regions, and consistency in response detection and reporting. The most important questions are based on the multidisciplinary and multi-dimensional aspect of data: how can we extract useful information in a shorter time and with a simpler representation? How reliable and reproducible are these results? How can we effectively implement and use them in the busy, time-sensitive workflow of the radiation oncology clinic?

The current literature suggests that combining these modalities through parametric response mapping (PRM) of the imaging data may be a more effective and efficient way of assessing tumor response [7,26-27]. The previously mentioned relationship between delivered radiation dosage, which is calculated the day of treatment, and treatment response, as calculated by Willowson et al. [15], needs further exploration. Comparing the estimated and calculated dosage delivery with PRMs may provide useful insights that could be used to better assess the relationship between delivered dosage and treatment response. This is suggested to promote the development of accurate and fast clinical post-treatment methods and platforms for more personalized treatment and better tumor response.

Identifying the types of patients that benefit the most and least from TARE is another area that needs further exploration. Pretreatment bio-markers for predicting success and/or failure should improve the patient selection process and prevent unnecessary treatments. Large, detailed population studies are needed to identify such bio-markers. Understanding who benefits from TARE and similar treatments, like TACE, is vital in determining the treatment plans of patients with liver malignancies.

## Conclusions

TARE treatment assessment can be improved using parametric response mapping (PRM), but most treatment success indicators, such as necrosis, change in size, vascularity, FDG-PET metabolic activity, and ADC cannot be identified within the first month after treatment. Treatment response needs to be determined as soon as possible to allow patients to receive an optimal therapy plan. Dosage delivery may have the potential to give early insight into treatment response. Treatment plans need to be further optimized by understanding what types of patients benefit most from TARE. The current imaging modalities are constantly being improved, and many new functional MRI techniques are being developed, such as glucoCEST, MR fingerprinting, and MR elastography. More research into how these and other novel methods of imaging can be better used for more accurate and faster tumor response assessment in TARE is needed to improve patient outcomes.
